# Status and correlates of home smoking bans after the implementation of the smoke-free legislation in public places: A survey in Chongqing

**DOI:** 10.18332/tid/146588

**Published:** 2022-05-03

**Authors:** Li Zhang, Zhiyong Zhang, Yang Cao, Ya Zhang, Mei Kuang, Yetao Luo, Li Jun, Yanhan Chen

**Affiliations:** 1 College of Nursing, Chongqing Medical University, Chongqing, People’s Republic of China; 2 Integrated Traditional Chinese Medicine and Western Medicine Department, Healthcare Center, Jinlong Town, People’s Republic of China; 3 Healthcare Center, Bafu Town, People’s Republic of China; 4 Nursing Department, Healthcare Center, Chongqing, People’s Republic of China; 5 Nursing Department, Township Hospitals, Jinfeng Town, People’s Republic of China; 6 Clinical Epidemiology and Biostatistics Department, Children’s Hospital, Chongqing Medical University, Chongqing, People’s Republic of China; 7 Chongqing Medical University, Chongqing, People’s Republic of China

**Keywords:** voluntary smoke-free rules, private places, smoke-free families, WHO FCTC, tobacco control resources

## Abstract

**INTRODUCTION:**

Secondhand smoke exposure in many countries decreased dramatically after the implementation of smoke-free legislation in public places, but the exposure at home did not change to the same degree. The aim of this study was to describe the status and correlates of a home smoking ban in Chongqing, China.

**METHODS:**

From June to August 2021, we selected two healthcare centers in the East, West, North, South and Middle regions of Chongqing. We investigated the family smoke-free situation in the selected region using a stratified random sampling method. A chi-squared test was performed to compare the totally and partially smoke-free homes, and a binary logistic regression model was used to analyze the correlates of smoke-free rules at home.

**RESULTS:**

The study investigated 2121 families, among which 884 (41.7%) implemented a total ban on smoking at home. The covariates included age (OR=1.54; 95% CI: 1.18–2.01), living with children aged <14 years (OR=1.51; 95% CI: 1.20–1.90), no smokers in the family (OR=2.37; 95% CI: 1.78–3.17), awareness of the hazards of secondhand smoke (OR=1.30; 95% CI: 1.07–1.59), worrying about the impact of smoking in the presence of children on health (OR=1.92; 95% CI: 1.25–2.95), no difficulty (OR=1.34; 95% CI: 1.07–1.67) and confidence (OR=1.73; 95% CI: 1.41–2.13) in stopping others from smoking, no smoking rules in cars (OR=3.67; 95% CI: 2.58–5.22), and frequency of entertaining guests with cigarettes (OR=0.41; 95% CI: 0.28–0.59).

**CONCLUSIONS:**

It is common for households in Chongqing to have smoking bans, especially those with children. If a family has members that are smokers, education researchers should pay more attention to the hazards of secondhand smoke on the health of family members, and to adopt more tobacco control measures and enhance the self-efficacy of implementing a home smoking ban. Helping smokers to quit is a vital way to decrease the hazards of cigarettes.

## INTRODUCTION

Tobacco kills more than 8 million people each year. Over 7 million of these deaths are caused by direct smoking, while about 1.2 million are the result of exposure of non-smokers to secondhand smoke^1^. Women and children are the main victims of secondhand smoke^2^, and public places and households are primary locations of exposure.

Effective interventions to reduce secondhand smoke exposure include smoke-free rules in work settings and public places, and the establishment and maintenance of smoke-free households. Article 8 of the World Health Organization (WHO) Framework Convention on Tobacco Control (FCTC) advocates and requires indoor workplaces and public places to be totally smoke-free^3^. In the last several decades, in response to the FCTC, a growing number of countries have introduced smoke-free legislation to eliminate secondhand smoke exposure in public places and workplaces. However, the household is regarded as a private place and is difficult to be regulated by the FCTC. Even if smoke-free public legislation becomes effectively universal, exposure to secondhand smoke at home remains a prominent issue, especially for vulnerable people such as children and women^4^.

China, the largest tobacco producer and consumer around the world, has 316 million current smokers with 68.1% of non-smokers being victims of secondhand smoke^5^. In order to reduce the prevalence of tobacco use, China signed the FCTC in 2003 and implemented a series of tobacco control laws, requiring that there be no smoking in public places, no tobacco advertising, smoke-free hospitals and schools, and leaders and cadres quitting smoking as role models^6^. These measures significantly reduced the exposure to secondhand smoke in public places^7,8^. Mothers more frequently protect their children from the hazards of secondhand smoking^9^. However, smoke-free legislation in public places led more smokers to believe that the household is a safe place for this behavior, in turn increasing the risk of family members’ exposure to secondhand smoke^10,11^.

As such, there is an urgent need for the Chinese government to control household indoor smoking, which is arguably the first priority to protect non-smokers in families from secondhand smoke exposure^12^. Unfortunately, the rate of home smoking bans was determined to be quite low, at only 6.3% in 2006 and 35.3% in 2014^12-16^, indicating that many women and children were still exposed to secondhand smoke, especially in rural areas^12,13^. Studies have suggested that about 18% of lung cancers in female non-smokers might be attributed to passive smoking, mainly caused by home exposure^17^.

Different areas in China experience different economic conditions, cultures and tobacco control legislation, resulting in different secondhand smoke exposure, among which the exposure rate of children in the southwest of China was found to be the highest^18^. Chongqing is a large municipality and a metropolis, but a large portion of its area comprises mountainous areas and large reservoir areas in southwestern China. It celebrated the first implementation of total tobacco control and smoke-free public places on 1 January 2021, but the status of home smoking bans was unknown at that time. Therefore, this study describes and discusses home smoking control and its correlates in Chongqing.

## METHODS

### Study design

From June to August 2021, we randomly selected two healthcare centers in the East, West, North, South and Middle regions of Chongqing, 10 healthcare centers in total, covering a population of about 520 thousand or 13–110 thousand in each center. Trained investigators explained the purpose of the survey and obtained informed consent when the respondents rested in the observation room after being injected the COVID-19 vaccine in community healthcare centers. Then, participants scanned a QR code with their smart phones and finished the questionnaire anonymously online. Those who had no smart phones filled in paper questionnaires. All the participants were given a pack of paper towels as a reward. The study was approved by the Ethics Committee of Chongqing Medical University.

The questionnaire was developed on the basis of the ‘knowledge–belief–practice’ model and informed by approaches in the literature^11-12,15-16,19^ after we consulted experts in: clinical tobacco control, public health, epidemiological statistics, community, and family management and sociology. The questionnaire was revised several times after it was pre-surveyed until the reliability and validity were verified, and it was ultimately used in the final survey. The Cronbach’s alpha coefficient was 0.81.

### Measures and questionnaire

Primary information included gender, age, education level, occupation, living with children aged <14 years, and current smoking status of family members. Current smokers we considered to be those who smoked at the time of the survey^20^.

Knowledge about tobacco control covered about 11 diseases that might be caused by secondhand smoke, as well as participants’ understanding of 5 tobacco control resources (tobacco control hotline, tobacco control applet (APP), tobacco control websites, tobacco control drugs, and tobacco control clinic). If an item was answered correctly, 1 point was recorded and the maximum possible score was 11. The options for the resources were from completely unknown to very familiar. Points ranged from 1–5 with a maximum total score of 25.

Family smoking control attitudes and self-efficacy were explored with 5 questions: ‘How important/ difficult do you think it is to ban smoking in your home?’, ‘How confident are you not to let others smoke in your home?’, ‘Are you worried that smoking in the presence of children would harm their health?’, and ‘Are you worried about your child smoking in the future?’. The answer was scored from 1 to 5 points ranging from not important at all /not difficult at all /not confident at all /not worried at all, to very important/very difficult/very confident/very worried.

Family smoking control rules were investigated with 4 items: ‘What are the smoking rules at your home besides smoking in the open balcony?’. Responses options were: smoking anywhere; in prescribed rooms; on special occasions such as family gatherings, guest visits, bad weather; and no smoking at my home. ‘What are the smoking rules in your car in the presence of children?’. Responses options were: no smoking; sometimes or in some cases; smoking; and no car. ‘How often do you entertain guests with cigarettes?’ and ‘How often do you give cigarettes as gifts?’. Response options were scored 1–5 from ‘never’ to ‘always’.

### Quality control

The questionnaire was developed after reviewing literature, and revised several times by experts and using pre-surveys until the reliability and validity were verified. In order to obtain only one response from a family, the questionnaire was sent to only one family member, and one IP address submitted once. The collected questionnaires were numbered and were input into the database by two persons independently. After repeatedly verified, they were used in the analysis.

### Data analysis

There is no safe threshold for secondhand smoking^2^, so only a totally smoke-free environment can protect family members from the harm of secondhand smoking. A household smoking control rule was explained as a total ban, i.e. no smoking anytime and anywhere except in the open balcony. Other conditions were defined as a total/partial ban such as smoking anywhere at home, smoking in some rooms and smoking in some cases, e.g. family gatherings, guest visits and bad weather.

Scores of tobacco control knowledge were divided into high and low groups. The possible responses regarding tobacco control attitude and self-efficacy were each re-coded into: ‘very important/important’ and ‘uncertain/somewhat important/not important at all’, ‘very difficult/difficult’ and ‘uncertain/somewhat difficult/not difficult at all’, ‘very confident/confident’ and ‘uncertain/a little confident/not confident at all’, ‘very worried/worried’ and ‘uncertain/a little worried/not worried at all’.

Data analysis was conducted in SPSS 20.0 (IBM Corporation, Armonk, NY, US). Descriptive analysis of participants’ characteristics used mean and standard deviation or composition ratio. Chi-squared test was used to detect the differences between the home tobacco ban rules. The significantly different factors in the chi-squared test were analyzed by binary logistic regression analysis. We controlled for : age, sex, family members smoking or not, and living with children aged <14 years. The threshold of statistical significance was set at 0.05.

## RESULTS

### Primary information

Questionnaire return was 78.4%. A total of 2121 participants completed the questionnaires, including 928 males (43.8%) and 1193 females (56.2%) with an average age of 36.30 ± 11.55 years. A total of 1123 (52.9%) participants had senior middle school or lower education, and 1256 (59.2%) were unemployed and lived in rural or urban areas. Among the participants, 1016 (47.9%) had smoker family members, and 1345 (73.4%) participants lived with children aged <14 years. A total of 884 (41.7%) had banned smoking anywhere at any time (i.e. no smoking at home), 659 (31.1%) restricted smoking at home during family gatherings, guest visits or bad weather; 404 (19.0%) allowed smoking in some rooms, and the remainder allowed smoking anywhere in the home.

### Basic characteristics of respondents and total home smoking ban

The overall smoking control rate of the female participants was higher than that of the male. Those who were aged 31–40 years administered home smoking control at a higher rate than other age groups. We found that 25.7% of families that had smoker members banned smoking, lower than those who had no smoker family members. And 47.1% of families who lived with children completely banned smoking, higher than those without children living at home. Therefore, the implementation of total smoking ban was related to gender, age, having smoker family members, and living with children aged <14 years. The difference was statistically significant (p<0.05) (Table 1).

**Table 1:** Characteristics of participants and total home smoking ban in Chongqing, China, 2021 (N=2121)

*Characteristics*	*Total*	*Total ban on smoking n (%)*	*Not total ban on smoking n (%)*	*χ* ^2^	*p*
**Total**	2121	884 (41.7)	1237 (58.3)		
**Gender**				10.66	<0.05
Male	928	350 (37.7)	578 (62.3)		
Female	1193	534 (44.8)	659 (55.2)		
**Age** (years)				43.05	<0.001
≤30	576	180 (31.2)	396 (68.8)		
31–40	879	419 (47.7)	460 (52.3)		
41–50	401	184 (45.9)	217 (54.1)		
≥51	265	101 (38.1)	164 (61.9)		
**Education level**				3.31	0.507
Primary school	195	78 (40.0)	117 (60.0)		
Middle school	427	173 (40.5)	254 (59.5)		
High middle school	501	206 (41.1)	295 (58.9)		
Junior college	408	163 (40.0)	245 (60.0)		
Undergraduate or above	590	264 (44.7)	326 (55.3)		
**Profession**				6.23	0.101
Director (government, enterprise or institution)	150	76 (50.7)	74 (49.3)		
Professional technician	367	151 (41.1)	216 (58.9)		
Service employee	348	135 (38.8)	213 (61.2)		
Farmer or unemployed	1256	522 (41.6)	734 (58.4)		
**Family members smoking status**				207.89	<0.001
I smoke	416	94 (22.6)	322 (77.4)		
Family members smoke	600	167 (27.8)	433 (72.2)		
No smokers in family	1105	623 (56.4)	482 (43.6)		
**Living with children younger than 14 years old**				45.07	<0.001
No	776	250 (32.2)	526 (67.8)		
Yes	1345	634 (47.1)	711 (52.9)		

### Smoking control knowledge, attitudes and complete home smoking ban

Participants’ secondhand smoke hazard knowledge scored from 0 to 11, with an average score of 7.10 ± 3.81. Participants’ scores on knowledge of tobacco control resources ranged from 5 to 23 with an average of 9.27 ± 3.47. The family whose tobacco control knowledge scored higher administered smoke-free rules better than those whose knowledge scored lower. A total of 1977 (93.2%) participants thought it was important to ban smoking at home, and 1933 (91.1%) participants worried that smoking in the presence of children endangered children’s health. In contrast, 760 (35.8%) participants responded that it was difficult to ban smoking at home. A total of 1029 (48.5%) participants were confident in their ability to prevent others from smoking in their households. Those participants set up more family smoke-free rules who thought smoking ban was very important, worried about smoking in the presence of children damaging the health of children, regarded family smoking ban not difficult, and who were confident to prevent others from smoke in their families. Smoking control knowledge, attitude and self-efficacy had impacts on the establishment of totally smoke-free homes. The differences were statistically significant (p<0.05) (Table 2).

**Table 2:** Smoking control knowledge, attitudes and total home smoking ban in Chongqing, China, 2021 (N=2121)

*Variables*	*Total n*	*Total ban on smoking n (%)*	*Not total ban on smoking n (%)*	*χ* ^2^	*p*
**Total**	2121	884 (41.7)	1237 (58.3)		
**Secondhand smoke hazard score**				26.87	<0.001
Low (1–7)	1012	363 (35.9)	649 (64.1)		
High (8–11)	1109	521 (47.0)	588 (53.0)		
**Knowledge score of tobacco control resources**				7.06	<0.05
Low (5–9)	1489	593 (39.8)	896 (60.2)		
High (10–25)	632	291 (46.0)	341 (54.0)		
**Are you worried about the impact of smoking on health in the presence of children?**				43.08	<0.001
No	188	36 (19.1)	152 (80.9)		
Yes	1933	848 (43.9)	1085 (56.1)		
**Are you worried about your children smoking in the future?**				10.07	<0.05
No	360	123 (34.2)	237 (65.8)		
Yes	1761	761 (43.2)	1000 (56.8)		
**Do you think it is important to ban smoking at home?**				54.11	<0.001
No	144	18 (12.5)	126 (87.5)		
Yes	1977	866 (43.8)	1111 (56.2)		
**Do you think it is difficult to ban smoking at home?**				80.62	<0.001
Yes	760	219 (28.8)	541 (71.2)		
No	1361	665 (48.9)	696 (51.1)		
**Are you confident to prevent others from smoking at home?**				161.31	<0.001
No	1092	311 (28.5)	781 (71.5)		
Yes	1029	573 (55.7)	456 (44.3)		

### Family smoking rule and custom, and total home smoking ban

A total of 1558 (73.5%) participants banned smoking in cars. We found that 684 (32.1%) had entertained guests with cigarettes, and 398 (18.8%) had given cigarettes as gifts. The family that banned smoking in cars, never or occasionally entertained guests with cigarettes, and never gave cigarettes as gifts, implemented smoke-free rules better than those who allowed smoking in cars, entertained guests with cigarettes and gave cigarettes as gifts. The differences were statistically significant (p<0.05) (Table 3).

**Table 3:** Family smoking rules and total home smoking ban in Chongqing, China, 2021 (N=2121)

*Variables*	*Total n*	*Total ban on smoking n (%)*	*Not total ban on smoking n (%)*	*χ* ^2^	*p*
**Total**	2121	884 (41.7)	1237 (58.3)		
**What are the smoking rules when children are in the car?**				145.36	<0.001
Smoking freely	328	45 (13.7)	283 (86.3)		
No car	235	78 (33.2)	157 (66.8)		
No smoking	1558	761 (48.8)	797 (51.2)		
**How often does your family entertain guests with cigarettes?**				156.92	<0.001
Never/occasionally	1437	723 (50.3)	714 (49.7)		
Sometimes	370	116 (31.4)	254 (68.6)		
Often/always	314	45 (14.3)	269 (85.7)		
**How often does your family give cigarettes as gifts?**				32.53	<0.001
Never/occasionally	1723	768 (44.6)	955 (55.4)		
Sometimes	295	90 (30.5)	205 (69.5)		
Often/always	103	26 (25.2)	77 (74.8)		

### Multifactorial analysis of total home smoking ban

Taking whether the home completely banned smoking (yes=1, no=0) as the dependent variable, and the 13 independent variables which were statistically significant (p<0.05) in univariate analysis (including demographics, smoking control knowledge, attitude, self-efficacy and home smoking rules), binary logistic regression analysis was conducted with the backward method. Meaningful results were found. The proportion of families of participants aged 30–40 years who established smoke-free homes was 1.54 times (95% CI: 1.18–2.01) that of families younger than 30 years. The proportion of families of participants with children aged <14 years who established smoke-free homes was 1.51 times (95% CI: 1.20–1.90) that without children <14 years. The proportion of families of participants without a smoker was 2.37 times (95% CI: 1.78–3.17) that of families with a smoker. The proportion of families of participants with higher scores of tobacco control knowledge was 1.30 times (95% CI: 1.07–1.59) that of families scoring lower. The proportion of families of participants worrying about the harm of smoking on children’s health was 1.92 times (95% CI: 1.25–2.95) that of families not worrying. The proportion of families of participants who were confident and had no difficulties preventing others from smoking in their families were 1.73 times (95% CI: 1.41–2.13) and 1.34 times (95% CI: 1. 07– 1.67), respectively, that of families not confident and found it difficult. The proportion of families of participants who totally banned smoking in their cars was 3.67 times (95% CI: 2.58–5.22) that of those with a partial ban. Participants who entertained guests with cigarettes found it not possible to ban smoking in their homes, and the proportion to set up smoke-free families was 0.41 times (95% CI: 0.28–0.59) that of those who did not entertain guests with cigarettes (Table 4).

## DISCUSSION

In order to determine the popularity and relevance of smoke-free home regulation, this study investigated the status of home smoking bans in Chongqing, China. This survey found that 41.7% of participants had completely banned smoking at home, which is a higher figure than that which has been reported in previous studies in Sichuan^13^, Shanghai^12,14^, Guangdong^15^ , and Guangxi^16^. This might be related to the continuous introduction of a series of tobacco control regulations and relevant tobacco control education in China in recent years. Additionally, 25.7% of smokers had completely banned smoking at homes, which was lower than the figure of 26.5% in six European countries^19^; 56.4% of the non-smoker families set up total smoke-free homes, obviously higher than that of the smoker families.

**Table 4:** Binary logistic regression analysis of total home smoking ban in Chongqing, China, 2021 (N=2121)

*Variables*	*b*	*S.E.*	*Wald*	*p*	*Exp (b)*	*95% CI*	
						*Lower*	*Lower Upper*
**Age** (years)			12.11	<0.05			
≤30 (Ref.)							
31–40	0.43	0.14	10.05	<0.05	1.54	1.18	2.01
41–50	0.39	0.16	6.23	<0.05	1.47	1.09	1.99
≥51	0.44	0.18	6.30	<0.05	1.56	1.10	2.20
**Are there children younger than 14 years old living with you?**							
No (Ref.)							
Yes	0.41	0.12	12.42	<0.001	1.51	1.20	1.90
**Family smoking status**			57.70	<0.001			
I smoke (Ref.)							
My family members smoke	0.09	0.16	0.30	0.59	1.09	0.79	1.50
No smoker in my family	0.86	0.15	34.32	<0.001	2.37	1.78	3.17
**Secondhand smoke hazard score**							
Low (Ref.)							
High	0.26	0.10	6.73	<0.05	1.30	1.07	1.59
**Are you confident to prevent others smoking at home?**							
No (Ref.)							
Yes	0.55	0.11	26.56	<0.001	1.73	1.41	2.13
**Are you worried about smoking impacting the health of children?**							
No (Ref.)							
Yes	0.65	0.22	8.87	<0.05	1.92	1.25	2.95
**Is it difficult to ban smoking in your home?**							
Yes (Ref.)							
No	0.29	0.11	6.60	<0.05	1.34	1.07	1.67
**Do you prevent smoking in your car?**			53.08	<0.001			
Partial (Ref.)							
No car	1.04	0.23	20.75	<0.001	2.84	1.81	4.45
Total	1.30	0.18	52.51	<0.001	3.67	2.58	5.22
**How often do you entertain guests with cigarettes?**			24.30	<0.001			
Never/Occasionally (Ref.)							
Sometimes	-0.30	0.14	4.85	<0.05	0.74	0.56	0.97
Often/always	-0.89	0.19	22.53	<0.001	0.41	0.28	0.59
**Constant**	-2.40	0.26	88.05	<0.001	0.09		

Adjusted for age, sex, family members smoking or not, living with children aged <14 years.

A smoke-free home can not only prevent exposure to secondhand smoke, but also improve the smoking cessation rate and reduce the cigarette consumption among adult smokers^21^. It can also establish and consolidate the values and rules of lifelong anti-smoking among teenagers, and reduce teenagers’ smoking behaviors^21,22^.

Knowledge of the serious harm of secondhand smoke has been reported to have a positive effect on smoking cessation^23,24^. The present study found that people who scored higher in the secondhand smoke hazard knowledge and who believed that secondhand smoke would cause serious harm to family members, especially children, were more likely to establish smoke-free homes. Participants, who were aged 30–40 years, and in families with children aged <14 years, and who were worried that smoking in the presence of children may cause damage to children’s health, were more likely to implement rules for complete ban on smoking. This might be related to Chinese culture, which is exhibits high collectivism in which the needs of groups precede those of individuals^23,25^. In the literature, it has been reported that many smokers know that smoking is harmful to their health, but tobacco dependence and the delay of harm presence leads them to insist on smoking, regardless of the health hazard. However, it has been reported when smokers perceive that their smoking poses a threat to the health of their families, the concept of collectivism and family responsibility promotes them to quit smoking in order to protect their family members and to develop smoke-free households, especially for the health of children^16,26,27^. Therefore, some smoke-free homes could be promoted by focusing on children’s health and by educating smokers about the harm of secondhand smoke to children, and encouraging smoking cessation for the sake of children’s health and setting examples for children^16,23^. Furthermore, pregnancy of the mother-to-be and childbirth were also found to be good opportunities to persuade smokers to quit smoking and achieve home smoking bans^28^.

The present study found that those who believed it was important to ban smoking at home, who felt it was not difficult to ban smoking at home and who were confident in preventing others from smoking at home, had a higher rate of implementing total bans on home smoking. This is consistent with the Health Belief Model. Smoking at home was found to be an obstacle to setting up a home smoking ban^12,13^. A lack of understanding and utilization of tobacco control resources might result in the failure of smoking cessation. The present study showed that people who did not have an understanding of tobacco control resources scored lower at an average of 9.269 ± 3.472 on the section of knowledge about tobacco control (maximum possible score of 25). Therefore, in the future, tobacco hazard education is necessary to communicate the serious harm of secondhand smoke and the benefits of smoke-free homes. Resources for smoking cessation^29,30^ should also be provided to help smokers to get appropriate cessation methods and to enhance self-efficacy, helping smokers to quit smoking and promoting the establishment of more smoke-free homes. Meanwhile, a smoke-free home can also stimulate smokers to quit smoking^21^. Therefore, in the presence of smoke-free public place regulation, an effective way to build smoke-free households is to provide smokers with smoking cessation resources and assistance for quitting smoking.

Offering cigarettes to guests or as gifts is a common social custom in many regions of China. Offering and accepting cigarettes is regarded as polite social behavior and interaction^31^. In this survey, 32.2% of respondents had previously entertained guests with cigarettes, and 18.8% had given cigarettes as gifts, suggesting that smoking was not perceived as a bad habit in Chongqing. Therefore, it is urgent to stress the use of education to change the social custom.

### Limitations

This study had some limitations. First, it was self-reported. Because of social expectation and the universal anti-smoking culture, there may have been some false reports. Second, the cross-sectional analysis excluded any inference of causality among the variables. Finally, the study only collected data in Chongqing. Although Chongqing is the largest municipality directly under the central government in Western China, it is possible that it does not represent the exposure of children to secondhand smoke at home across China.

## CONCLUSIONS

Knowledge of the harm of secondhand smoke was a major driver of total home smoking bans, which was hindered by factors such as the presence of smokers at home and the lack of self-efficacy. In the context of smoke-free public places regulation, homes should be focused in public strategies to strengthen the education of secondhand smoke harms and change the social customs of entertaining others with cigarettes and giving cigarettes as gifts. In addition, more smoking control resources, higher self-efficacy in establishing smoke-free homes, and helping smokers to quit smoking are important interventions to reduce the harm of tobacco.

## Data Availability

The data supporting this research are available from the authors on reasonable request.

## References

[1] World Health Organization (2019). WHO report on the global tobacco epidemic 2019: offer help to quit tobacco use.

[2] World Health Organization (2021). Tobacco.

[3] WHO Framework Convention on Tobacco Control Guidelines for implementation of Article 8.

[4] Abdullah AS, Driezen P, Sansone G (2014). Correlates of exposure to secondhand smoke (SHS) at home among non-smoking adults in Bangladesh: findings from the ITC Bangladesh survey. BMC Pulm Med.

[5] World Health Organization Global Adult Tobacco Survey Fact Sheet.

[6] Yang G, Wang Y, Wu Y, Yang J, Wan X (2015). The road to effective tobacco control in China. Lancet.

[7] Duan Z, Wang Y, Huang J, Redmon PB, Eriksen MP (2020). Secondhand smoke (SHS) exposure before and after the implementation of the Tobacco Free Cities (TFC) initiative in five Chinese cities: a pooled cross-sectional study. BMJ Open.

[8] Xiao L, Jiang Y, Liu X, Li Y, Gan Q, Liu F (2017). Smoking reduced in urban restaurants: the effect of Beijing Smoking Control Regulation. Tob Control.

[9] Chan SS, Cheung YT, Leung DY, Mak YW, Leung GM, Lam TH (2014). Secondhand smoke exposure and maternal action to protect children from secondhand smoke: pre-and post-smokefree legislation in Hong Kong. PLoS One.

[10] Ho SY, Wang MP, Lo WS (2010). Comprehensive smoke-free legislation and displacement of smoking into the homes of young children in Hong Kong. Tob Control.

[11] Zheng ZL, Deng HY, Wu CP (2017). Secondhand smoke exposure of children at home and prevalence of parental smoking following implementation of the new tobacco control law in Macao. Public Health.

[12] Zheng P, Kegler MC, Berg CJ (2014). Correlates of smoke-free home policies in Shanghai, China. Biomed Res Int.

[13] Wang CP, Ma SJ, Xu XF, Wang JF, Mei CZ, Yang GH (2009). The prevalence of household second-hand smoke exposure and its correlated factors in six counties of China. Tob Control.

[14] Ji M, Ding D, Hovell MF, Xia X, Zheng P, Fu H (2009). Home smoking bans in an urbanizing community in China. Am J Prev Med.

[15] Wei X, Zhang Z, Song X (2014). Household smoking restrictions related to secondhand smoke exposure in Guangdong, China: a population representative survey. Nicotine Tob Res.

[16] Huang K, Chen H, Liao J (2016). Factors Associated with Complete Home Smoking Ban among Chinese Parents of Young Children. Int J Environ Res Public Health.

[17] Du Y, Cui X, Sidorenkov G (2020). Lung cancer occurrence attributable to passive smoking among never smokers in China: a systematic review and meta-analysis. Transl Lung Cancer Res.

[18] Xie M, Jia C, Zhang Y (2020). Household Exposure to Secondhand Smoke among Chinese Children: Status, Determinants, and Co-Exposures. Int J Environ Res Public Health.

[19] Fu M, Castellano Y, Tigova O (2019). Prevalence and correlates of different smoking bans in homes and cars among smokers in six countries of the EUREST-PLUS ITC Europe Surveys. TobInduc Dis. Tob Induc Dis.

[20] World Health Organization 2018 China Adult Tobacco Survey Report.

[21] Mills AL, Messer K, Gilpin EA, Pierce JP (2009). The effect of smoke-free homes on adult smoking behavior: a review. Nicotine Tob Res.

[22] Emory K, Saquib N, Gilpin EA, Pierce JP (2010). The association between home smoking restrictions and youth smoking behaviour: a review. Tob Control.

[23] Madewell ZJ (2018). The belief that secondhand smoke causes serious illness among Chinese smokers: Smoking cessation and intention to quit. Tob Prev Cessat.

[24] Song AV, Glantz SA, Halpern-Felsher BL (2009). Perceptions of second-hand smoke risks predict future adolescent smoking initiation. J Adolesc Health.

[25] Mao A, Bottorff JL, Oliffe JL, Sarbit G, Kelly MT (2018). A Qualitative Study on Chinese Canadian Male Immigrants’ Perspectives on Stopping Smoking: Implications for Tobacco Control in China. Am J Mens Health.

[26] Mao A, Bottorff JL, Oliffe JL, Sarbit G, Kelly MT (2015). A qualitative study of Chinese Canadian fathers’ smoking behaviors: intersecting cultures and masculinities. BMC Public Health.

[27] Yang T, Fisher KJ, Li F, Danaher BG (2006). Attitudes to smoking cessation and triggers to relapse among Chinese male smokers. BMC Public Health.

[28] Fu C, Chen Y, Wang T, Edwards N, Xu B (2008). Exposure to environmental tobacco smoke in Chinese new mothers decreased during pregnancy. J Clin Epidemiol.

[29] Zhang L, Li J, Lv Y (2020). Impact of tobacco control auxiliary resources on the 5As behavior in nursing interns: Self-reports from students. Tob Induc Dis.

[30] Lin H, Xiao D, Liu Z, Shi Q, Hajek P, Wang C (2019). National survey of smoking cessation provision in China. Tob Induc Dis.

[31] Hu M, Rich ZC, Luo D, Xiao S (2012). Cigarette sharing and gifting in rural China: a focus group study. Nicotine Tob Res.

